# Cost-effectiveness Analysis of the Elder-Friendly Approaches to the Surgical Environment (EASE) Intervention for Emergency Abdominal Surgical Care of Adults Aged 65 Years and Older

**DOI:** 10.1001/jamanetworkopen.2020.2034

**Published:** 2020-04-03

**Authors:** Mark Hofmeister, Rachel G. Khadaroo, Jayna Holroyd-Leduc, Raj Padwal, Adrian Wagg, Lindsey Warkentin, Fiona Clement

**Affiliations:** 1 Department of Community Health Sciences, University of Calgary, Calgary, Alberta, Canada; 2 O’Brien Institute for Public Health, University of Calgary, Calgary, Alberta, Canada; 3 Department of Surgery, University of Alberta, Edmonton, Alberta, Canada; 4 Department of Medicine, University of Calgary, Calgary, Alberta, Canada; 5 Department of Medicine, University of Alberta, Edmonton, Alberta, Canada

## Abstract

**Importance:**

The Elder-Friendly Approaches to the Surgical Environment (EASE) initiative is a novel approach to acute surgical care for elderly patients.

**Objective:**

To determine the cost-effectiveness of EASE.

**Design, Setting, and Participants:**

An economic evaluation from the perspective of the health care system was conducted as part of the controlled before-and-after EASE study at 2 tertiary care centers, the University of Alberta Hospital and Foothills Medical Centre. Participants included elderly adults (aged ≥65 years) admitted for emergency abdominal surgery between 2014 and 2017. Data were analyzed from April 2018 to February 2019.

**Main Outcomes and Measures:**

Data were captured at both control and intervention sites before and after implementation of the EASE intervention. Resource use was captured over 6 months of follow-up and was converted to costs. Utility was measured with the EuroQol Five-Dimensions Three-Levels instrument at 6 weeks and 6 months of follow-up. The differences-in-differences method was used to estimate the association of the intervention with cost and quality-adjusted life-years. For a subset of participants, self-reported out-of-pocket health care costs were collected using the Resource Use Inventory at 6 months.

**Results:**

A total of 675 participants were included (mean [SD] age, 75.3 [7.9] years; 333 women [49.3%]), 289 in the intervention group and 386 in the control group. The mean (SD) cost per control participant was $36 995 ($44 169) before EASE and $35 032 ($43 611) after EASE (all costs are shown in 2018 Canadian dollars). The mean (SD) cost per intervention participant was $56 143 ($74 039) before EASE and $39 001 ($59 854) after EASE. Controlling for age, sex, and Clinical Frailty Score, the EASE intervention was associated with a mean (SE) cost reduction of 23.5% (12.5%) (*P* = .02). The change in quality-adjusted life-years observed associated with the intervention was not statistically significant (mean [SE], 0.00001 [0.0001] quality-adjusted life-year; *P* = .72). The Resource Use Inventory was collected for 331 participants. The mean (SE) odds ratio for having 0 out-of-pocket expenses because of the intervention, compared with having expenses greater than 0, was 15.77 (3.37) (*P* = .02). Among participants with Resource Use Inventory costs greater than 0, EASE was not associated with a change in spending (mean [SE] reduction associated with EASE, 19.1% [45.2%]; *P* = .57).

**Conclusions and Relevance:**

This study suggests that the EASE intervention was associated with a reduction in costs and no change in quality-adjusted life-years. In locations that lack capacity to implement this intervention, costs to increase capacity should be weighed against the estimated costs avoided.

## Key Points

**Question:**

From the perspective of the health care system, what are the economic outcomes of the Elder-Friendly Approaches to the Surgical Environment intervention?

**Findings:**

In this economic evaluation of a controlled before-and-after study of elderly Canadian adults admitted for emergency abdominal surgery, the Elder-Friendly Approaches to the Surgical Environment intervention was associated with a reduction in costs and no change in quality-adjusted life-years.

**Meaning:**

These findings suggest that implementation of the Elder-Friendly Approaches to the Surgical Environment intervention should be considered in other centers; in locations lacking current capacity to implement this intervention, costs to increase capacity should be weighed against the estimated costs avoided.

## Teaser

This economic evaluation study examines the cost-effectiveness of the Elder-Friendly Approaches to the Surgical Environment (EASE) initiative for Canadian adults aged 65 years and older admitted for emergency abdominal surgery.

## Introduction

In 2014, 15.6% of the Canadian population was aged 65 years or older, and this proportion is expected to increase to 23% by 2030.^1^ Age has been identified as an independent variable associated with surgical mortality and morbidity and has been associated with increased cost.^2,3^ This is primarily associated with the increased prevalence of frailty with age.^4^

If age-specific use of surgical services remains constant, an aging population will result in a substantially increased demand for surgical services and health care costs.^5^ The hospital environment also subjects the older adult to increased risk of depression, delirium, and functional decline,^6,7,8^ which may not improve after hospital discharge.^7,9^ Therefore, measures to address hospitalization-related risks and surgical complications among older adults, as well as their associated costs, are critical.

The Acute Care for the Elderly (ACE) model of care is an initiative developed to address some of the risks associated with hospitalization for older patients. The ACE model promotes practices of specialized patient environments, patient-centered care, medical review, and interdisciplinary team plans of care in order to prevent the commonly observed functional decline among older hospitalized medical patients.^10^ The use of ACE models in medical wards has been associated with decreased cost and length of hospital stay, fewer readmissions, and improved cognition, function, and satisfaction for both patients and staff.^10,11,12^ The Elder-Friendly Approaches to the Surgical Environment (EASE) initiative (ClinicalTrials.gov identifier NCT02233153) represents a novel implementation of the ACE model for acute surgical patients. The EASE initiative is a multicomponent intervention designed to enhance perioperative elderly care through capacity realignment, multidisciplinary care teams that include geriatricians, evidence-informed elder-friendly practices, functional reconditioning, and transition optimization.^13^

This analysis describes findings from an economic evaluation of the EASE initiative. An economic evaluation was incorporated a priori into the overall study to assess the value of the intervention.^13^ Contributing to the complete reporting of all preestablished EASE study outcomes, the objective of this study is to complete an economic evaluation of EASE interventions for elderly patients recovering from emergency abdominal surgery, from the perspective of the health care system.

## Methods

### Enrollment and Follow-up

The EASE study was a concurrently controlled before-and-after study at 2 tertiary care hospitals in Canada, which enrolled participants from 2014 to 2017. The EASE study was implemented at the University of Alberta Hospital (Edmonton, Alberta), and a comparable site, the Foothills Medical Centre (Calgary, Alberta), served as a control against temporal changes. Both hospitals are tertiary care centers and serve similar patient populations. After a period of preintervention data collection at both sites (before EASE), the EASE initiatives were implemented at the intervention site, and a comparable site with approximately the same patient intake volume and acuity level had no implementation and served as the control site (after EASE). Older adults, aged 65 years or older, admitted for emergency abdominal surgery at either study site were considered for enrollment. Only index admissions were eligible for inclusion. Readmissions, elective or trauma surgery cases, palliative interventions, and transfers from another jurisdiction or other hospital services were excluded. Patients with preoperative dependency in 3 or more activities of daily living were ineligible for study inclusion. Demographic information, including the revised Canadian Study of Health and Aging Clinical Frailty Score,^14^ and in-hospital preoperative, perioperative, and postoperative details were collected through medical record review.

 Before study enrollment, all participants provided written informed consent. Study participants also provided written informed consent for follow-up data collection after discharge. All EASE study protocols have been reviewed and approved at both study sites (University of Alberta Health Research Ethics Board and University of Calgary Conjoint Research Ethics). This study follows the Consolidated Health Economic Evaluation Reporting Standards (CHEERS) reporting guideline.

### Economic Evaluation

The perspective of a publicly funded health care system was taken. The primary outcome is the incremental cost-effectiveness ratio. Costs are calculated over 6 months of follow-up. Quality-adjusted life-years (QALYs) were calculated using the area-under-the-curve method with EuroQol Five-Dimensions Three-Levels (EQ-5D-3L) measurements at 4 to 8 weeks (collectively referred to as 6 weeks) and 6 months after discharge. In a secondary analysis, a broader perspective was adopted, and patient-borne costs were included.

### Resource Use and Costs

Resource use was captured through individual, patient-level data in administrative databases for 6 months after enrollment. Index admissions and subsequent hospital admissions were captured in the Discharge Abstract Database, and in-hospital microcosting data were available from each site. For admissions with both microcosting data and Discharge Abstract Database data, microcosting was used preferentially. To calculate the costs of admissions captured in the Discharge Abstract Database only, resource intensity weight was multiplied by the cost of a standard hospital stay to arrive at the cost per episode of care. Ambulatory care was captured in the National Ambulatory Care Reporting System. Again, resource intensity weight was multiplied by the cost of a standard hospital stay to arrive at the cost per episode of care. Where the cost of a standard hospital stay was required for Discharge Abstract Database and National Ambulatory Care Reporting System observations, ordinary least-squares regression was fit to all available previous cost of a standard hospital stay to estimate values where data were not yet available. Physician billing and prescription drug costs were also included. The drug identification number for each prescription dispensed was used to identify the appropriate unit cost, which was multiplied by the number of units dispensed. The 2018 Alberta Health Drug Benefit List was used to identify the appropriate unit cost for all prescriptions dispensed.^15^ Where appropriate, costs have been inflated to 2018 Canadian dollars using the consumer price index.^16^ All costs are presented in 2018 Canadian dollars (using 2018 purchasing power parity, 1 CAD$ = $0.83 US). Because outcomes were calculated over 6 months for each participant, no discount rate was applied.

### Utility Scores

Health-related quality of life was captured with the EQ-5D-3L instrument at 6 weeks and 6 months after discharge. The EQ-5D-3L instrument assesses self-reported health-related quality of life across 5 dimensions: mobility, self-care, usual activities, pain or discomfort, and anxiety or depression.^17^ Within each dimension, respondents indicate whether they have no impairment, some impairment, or complete impairment.^17^ The EQ-5D-3L index scores were converted to utility using the Canadian time trade-off scoring algorithm.^18^ Observed QALYs were calculated as area under the curve from 6 weeks to 6 months after discharge. For those participants missing 1 EQ-5D-3L response, the last observation carried forward was used to impute missing data. In this analysis, the last observation carried forward imputation assumes that in the absence of additional information, a change in utility is unlikely and avoids the loss of power associated with complete case analysis.^19^

### Statistical Analysis

 Data were analyzed from April 2018 to February 2019. For all analyses, statistical significance was set at 2-sided α = .05. All analyses were performed with R statistical software version 3.5.3 (R Project for Statistical Computing). The differences-in-differences (DD) method was used to analyze the association of the EASE intervention with cost and utility outcomes.^20^ The DD method requires a comparison group that experiences the same trends but was not exposed to the intervention.^20^ In the linear model used to calculate the DD, *Y_i_ = *α* + *β_1_*d_site_ + *β_2_*d_intervention_ + *β_3_d_site*intervention_ + X_i_, where *Y_i_* is the outcome of interest; α is the constant term; β indicates regression coefficients; *d_site_* is a dummy variable for site, with 1 indicating the intervention site and 0 indicating the control site; *d_intervention_* is a dummy variable for the EASE intervention, with 0 indicating before EASE and 1 indicating after EASE; d_site* intervention_ is the interaction term between the site and intervention variables; and *X_i_* is a vector of controls, including age, sex, and Clinical Frailty Score. Control variables were selected on the basis of clinical relevance. Age has been identified as an independent variable associated with surgical mortality and morbidity and has been associated with increased cost.^2,3^ Frailty describes a combination of disease, functional ability, and other factors, such as nutritional status.^4^ Frailty was also selected as a control variable, to limit the impact of these features on outcomes associated with the EASE intervention. In the regression equation, the interaction term between the site and intervention variables is the DD. Cost outcomes were log-transformed before analysis with the DD method to reduce skewness and improve linearity of the association between cost and independent variables. The QALYs observed were left-skewed and required a square root transformation to reduce skewness.

#### Incremental Cost-effectiveness Ratio

Costs and QALYs at each site are calculated as those before EASE minus those after EASE. To calculate incremental costs and QALYs, pre-EASE outcomes were subtracted from post-EASE outcomes. To calculate the incremental cost-effectiveness ratio, incremental costs were divided by incremental QALYs to arrive at the additional cost to achieve 1 QALY.

#### Secondary Analysis

For a subset of study participants, self-reported costs through the Resource Utilization Inventory (RUI) were collected to capture out-of-pocket patient and caregiver costs incurred in the 6 months after discharge. The RUI has been previously validated.^21^ Where approved benefits through the Alberta Aids to Daily Living program existed, these costs were attached to RUI responses^22^ (eTable in the Supplement). The average hourly wage in Alberta in 2017 was $28.39,^23^ which was inflated to 2018 Canadian dollars at $29.09^24^ and was used to estimate costs for participant time and informal care.

To analyze cost data collected in the RUI, a 2-stage model was used. This method was selected to accommodate data in which a substantial proportion of the values are 0 and the remainder of values have a skewed distribution.^25^ The RUI cost data are split into 2 data sets from the original: one indicating whether costs were 0, and the other indicating the logarithm of the cost when it was present. The presence of 0 cost observations was modeled with logistic regression, and the log-cost data were modeled with the DD method previously described.

## Results

### Participants

**Figure 1.**
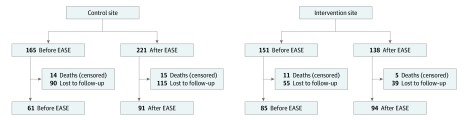
Participant Flow Diagram The 675 participants included in the analyses were identified through index admission, and their resource use data were captured. Of those patients, 331 completed the Resource Use Inventory and EuroQol Five-Dimensions Three-Levels instrument at 6 months of follow-up. EASE indicates Elder-Friendly Approaches to the Surgical Environment.

A total of 684 participants were identified through an index admission; however, 9 participants were lost to follow-up before index admission cost data were collected and were excluded from this analysis. Six hundred seventy-five participants were included in this analysis: 289 in the intervention group and 386 in the control group (mean [SD] age, 75.3 [7.9] years; 333 women [49.3%]) (Figure 1). At the time of enrollment, study participants were comparable in terms of age (mean [SD] age, 75.5 [7.8] years at the control site vs 75.0 [8.0] years at the intervention site), sex (189 women [49.0%] at the control site vs 144 women [49.8%] at the intervention site), and Clinical Frailty Score (mean [SD], 3.7 [1.1] at the control site vs 4.0 [1.4] at the intervention site) (Table 1). Similar ages and proportions of each sex were observed before and after the intervention. The most common admitting diagnoses were cholecystitis or cholelithiasis (175 patients [25.9%]) and gastrointestinal obstruction not due to hernia (146 patients [21.6%]).

**Table 1.** Participant Characteristics at Enrollment

Characteristic	Participants, No. (%)			
	Control site		Intervention site	
	Before EASE (n = 165)	After EASE (n = 221)	Before EASE (n = 151)	After EASE (n = 138)
Age, mean (SD), y	76.3 (8.0)	74.9 (7.7)	75.3 (8.2)	74.6 (7.7)
Age group, y				
<65	1 (0.6)	0	0	0
≥65 to <75	79 (47.9)	119 (53.8)	79 (52.3)	80 (58.0)
≥75 to <85	58 (35.2)	69 (31.2)	48 (31.8)	40 (29.0)
≥85	27 (16.4)	33 (14.9)	24 (15.9)	18 (13.0)
Female	82 (50.0)	107 (48.4)	71 (47.0)	73 (52.9)
Clinical Frailty Score, mean (SD)	4.1 (1.2)	3.4 (1.0)	4.6 (1.4)	3.3 (1.3)
1	4 (2.4)	1 (0.5)	11 (7.3)	14 (10.1)
2	23 (13.9)	39 (17.6)	33 (21.9)	24 (17.4)
3	61 (37.0)	93 (42.1)	46 (30.5)	50 (36.2)
4	40 (24.2)	55 (24.9)	21 (13.9)	23 (16.7)
5	23 (13.9)	30 (13.6)	18 (11.9)	18 (13.0)
6	14 (8.5)	3 (1.4)	22 (14.6)	9 (6.5)
Admitting diagnosis category				
Appendix	19 (11.5)	35 (15.8)	14 (9.3)	11 (8.0)
Cancer	6 (3.6)	15 (6.8)	9 (6.0)	11 (8.0)
Cholecystitis or cholelithiasis	44 (26.7)	64 (29)	25 (16.6)	42 (30.4)
Diverticular	4 (2.4)	3 (1.4)	8 (5.3)	3 (2.2)
Hernia	22 (13.3)	26 (11.8)	28 (18.5)	17 (12.3)
Infection, inflammation, or ischemia	10 (6.1)	15 (6.8)	7 (4.6)	4 (2.9)
Gastrointestinal obstruction (not hernia)	44 (26.7)	33 (14.9)	34 (22.5)	35 (25.4)
Pancreas	5 (3.0)	12 (5.4)	2 (1.3)	2 (1.4)
Peritonitis	6 (3.6)	10 (4.5)	15 (9.9)	8 (5.8)
Other	5 (3.0)	8 (3.6)	9 (6.0)	5 (3.6)
American Society of Anesthesiologists physical status classification, mean (SD)	2.55 (0.76)	2.63 (0.93)	2.93 (0.77)	2.7 (0.67)
Charlson Comorbidity Index score, mean (SD)	0.94 (1.28)	1.13 (1.42)	1.28 (1.62)	1.01 (1.34)

Abbreviation: EASE, Elder-Friendly Approaches to the Surgical Environment.

At the control site, the numbers of deaths before and after EASE were similar (14 participants [8.5%] and 15 participants [6.8%], respectively). At the intervention site, 11 deaths (7.3%) were observed before EASE and 5 deaths (3.6%) were observed after EASE. Among participants who died, the mean age and Clinical Frailty Scores were similar between sites at each time point. Thirty-four participants (5.0%) lacked 1 EQ-5D-3L measurement, and responses for these participants were imputed.

### Resource Use and Costs

The mean (SD) total cost per participant decreased over the course of 6 months from $36 995 ($44 169) to $35 032 ($43 611) at the control site, and from $56 143 ($74 039) to $39 001 ($59 854) at the intervention site (Table 2). At the control site, the mean (SD) index admission costs per participant were $23 034 ($34 762) before EASE and $24 910 ($37 285) after EASE. The mean (SD) cost of index admission at the intervention site was $33 265 ($57 722) before EASE and $25 583 ($50 893) after EASE. At the control site, the mean (SD) number of readmissions per participant were 0.3 (0.7) before EASE and 0.2 (0.5) after EASE. At the intervention site, the mean (SD) number of readmissions per participant were 0.5 (1.0) before EASE and 0.4 (0.9) after EASE. The mean (SD) length of stay per readmission decreased from 13.7 (25.5) days to 11.8 (15.1) days at the control site and from 13.8 (19.7) days to 7.5 (10.5) days at the intervention site. Among those with readmissions, the mean (SD) cost per readmission decreased from $17 667 ($31 901) to $15 350 ($15 310) at the control site and from $19 631 ($29 008) to $11 476 ($17 478) at the intervention site. The mean number of visits per participant to ambulatory care and the mean costs associated with ambulatory care were similar between sites and time points. The mean (SD) cost per participant for medications decreased from $1156 ($5247) before EASE to $888 ($3361) after EASE at the control site, and from $801 ($1396) before EASE to $751 ($1735) after EASE at the intervention site. The mean (SD) costs per participant for physician services at the control site were $4196 ($3394) before EASE and $4050 ($3991) after EASE. The mean (SD) costs per participant for physician services at the intervention site were $6893 ($7700) before the intervention and $5357 ($5778) after the intervention. Controlling for sex, age, and Clinical Frailty Score, the EASE intervention was associated with a mean (SE) cost reduction of 23.5% (12.5%) (*P* = .02) among all enrolled participants.

**Table 2.** Observed Resource Use and Costs^a^

Variable	Mean (SD)			
	Control site		Intervention site	
	Before EASE (n = 165)	After EASE (n = 221)	Before EASE (n = 151)	After EASE (n = 138)
Index admission				
Length of stay, d	10.9 (16.5)	10.9 (13.7)	14.9 (18.0)	13.4 (27.6)
Cost for index admission, CAD$	23 034 (34 762)	24 910 (37 285)	33 265 (57 722)	25 583 (50 893)
Readmissions per participant, No.	0.3 (0.7)	0.2 (0.5)	0.5 (1.0)	0.4 (0.9)
Length of stay per readmission, d	13.7 (25.5)	11.8 (15.1)	13.8 (19.7)	7.5 (10.5)
Cost per readmission, CAD$	17 667 (31 901)	15 350 (15 310)	19 631 (29 008)	11 476 (17 478)
Cost per participant for readmissions, CAD$	5782 (21 000)	2570 (9806)	12 090 (34 387)	4657 (16 318)
Ambulatory care visits per participant, No.	5.6 (9.1)	4.7 (8.2)	5.9 (8.7)	5.3 (7.2)
Cost per ambulatory care visit, CAD$	508 (492)	561 (463)	528 (1211)	502 (698)
Cost per participant for ambulatory care visits, CAD$	2827 (4373)	2614 (4966)	3093 (5353)	2654 (3125)
Prescriptions dispensed per participant, No.	13.2 (16.2)	13.5 (21.4)	25.3 (48.3)	18.1 (28.4)
Cost per dispensation, CAD$	87.4 (702)	65.6 (685)	31.7 (140)	39.8 (223)
Cost per participant for medications, CAD$	1156 (5247)	888 (3361)	801 (1396)	751 (1735)
Cost per participant for physician services, CAD$	4196 (3394)	4050 (3991)	6893 (7700)	5357 (5778)
Total cost per participant, CAD$	36 995 (44 169)	35 032 (43 611)	56 143 (74 039)	39 001 (59 854)

Abbreviations: EASE, Elder-Friendly Approaches to the Surgical Environment.

^a^ All costs are given in 2018 Canadian dollars. Using 2018 purchasing power parity, 1 CAD$ = $0.83 US.

### Utility Scores

**Figure 2.**
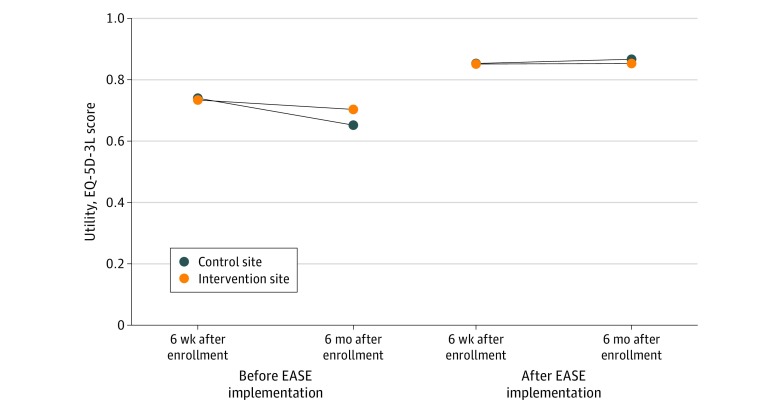
Utility, as Measured With the EuroQol Five-Dimensions Three-Levels (EQ-5D-3L) Instrument and Converted With the Canadian Scoring Algorithm EASE indicates Elder-Friendly Approaches to the Surgical Environment.

At the control site, the mean (SD) QALY observed per participant was 0.325 (0.063) before EASE implementation and was 0.332 (0.040) after implementation of EASE. At the intervention site, the mean (SD) QALY observed per participant was 0.320 (0.058) before implementation of the intervention and was 0.329 (0.056) after implementation of the intervention (Figure 2). The change in QALYs observed associated with the intervention was not statistically significant (mean [SE], 0.00001 [0.0001] QALY; *P* = .72).

### Incremental Cost-effectiveness Ratio

The mean cost per participant at the intervention site was $15 179 lower than at the control site (calculated as after EASE minus before EASE, at each site). Because the intervention site had greater QALYs observed at a reduced cost, the EASE intervention dominated the control. Density plots of QALYs and costs are included in the eFigure in the Supplement.

### Secondary Analysis

Costs to participants are included in Table 3. Of the 331 participants for whom RUI inventory data were collected, 29 participants had costs of 0. Of the participants who had no costs in the RUI, 22 were at the control site before EASE, 3 were at the control site after EASE, 2 were at the intervention site before EASE, and 2 were at the intervention site after EASE. Before EASE, the mean (SD) cost per participant was $5091 ($9871) at the control site and $6013 ($6812) at the intervention site. After EASE, the mean (SD) cost per participant was $5458 ($9256) at the control site and $8115 ($15 808) at the intervention site. The mean (SE) odds ratio of having 0 expenses captured by the RUI because of the EASE intervention, compared with having expenses greater than 0, was 15.77 (3.37) (*P* = .02) controlling for age, sex, and Clinical Frailty Score. Among those participants with RUI costs greater than 0, the EASE intervention was not associated with a statistically significant change in spending (mean [SE] reduction associated with EASE, 19.1% [45.2%]; *P* = .57).

**Table 3.** Resource Use Inventory Patient Expenses

Variable	Cost, mean (SD), $^a^			
	Control site		Intervention site	
	Before EASE	After EASE	Before EASE	After EASE
Aids to activities of daily living	562 (1489)	1653 (1483)	1286 (1376)	2070 (3393)
Informal care	3221 (7614)	2147 (4977)	3893 (5755)	3996 (10 500)
Lost hours	1308 (4805)	2024 (7024)	468 (2823)	2049 (7756)
Total patient cost	5091 (9871)	5458 (9256)	6013 (6812)	8115 (15 808)

Abbreviation: EASE, Elder-Friendly Approaches to the Surgical Environment.

^a^ All costs are given in 2018 Canadian dollars. Using 2018 purchasing power parity, 1 CAD$ = $0.83 US.

## Discussion

From the health care system perspective, the EASE initiative was associated with a substantial reduction in costs, with no evidence of a difference in QALY outcomes. Costs attached to self-reported RUI responses suggested that patients and their caregivers were less likely to spend money on health care when EASE interventions were implemented. These findings indicate that elder-friendly perioperative care may reduce costs to the health care system, without increasing costs to patients and without detriment to effectiveness measured in QALYs.

The context of the EASE study^13^ provided an ideal laboratory for economic evaluation. The DD method assumes that in the absence of the intervention, the unobserved differences between control and intervention groups are the same over time. The tertiary care centers selected as intervention and control sites in this study were selected because they had similar patient populations served in terms of volume, acuity, and proximity to university medical schools. Alberta stands out among Canadian provinces for having the largest provincewide, fully integrated health system.^26^ Provincial coordination of health care services reduces heterogeneity in care provided and supports the main assumption of DD. Furthermore, provincial collection of administrative data used to inform costing eliminates loss of data that may occur because of differences in data collection across other health systems.

Interestingly, mean costs per participant at the intervention site were greater than those at the control site at each time point, which suggests the presence of unmeasured systematic differences between the 2 sites. In this study, data were collected from 2 groups at 2 time points, with 1 group at the last time point receiving the intervention. This is the simplest setting for use of the DD method and, in this case, is equivalent to the 2-way fixed-effects approach with treatment and time indicators.^27^ If a greater number of study sites had been included, the 2-way fixed-effects approach could have been used to reduce the influence of preperiod-level differences on estimates of effect. Additional work is required to understand differences between the 2 sites and to take advantage of cost-reducing practices occurring already at the control site.

In a systematic review by Ahmed and Pearce,^10^ 6 studies reported costs of ACE models of care. In 2 of these studies, a neutral outcome on cost was reported.^10^ In the remaining 4 studies, the ACE model was associated with a decrease in total costs, hospital costs, or cost of care.^10^ This evaluation of an ACE model in the surgical setting supports the generalization of cost reductions identified in previous ACE studies to the setting of emergency abdominal surgical patients.

### Limitations

This study has several limitations. First, this was not a randomized clinical trial, which limits the ability to infer causation. Second, the RUIs are affected by a substantial loss to follow-up, which reduces the power to detect effects of EASE interventions. Compared with RUI responders, those who were lost to follow-up had much greater mean clinical frailty scores and greater mean age, which likely contributed to patient health care spending. However, the comparable numbers of participants lost to follow-up at each site and at each time point reduces the potential impact of this limitation. Third, implementation costs associated with the EASE interventions were not included in this analysis; therefore, initial costs of care cannot be commented on. In settings that already have the network of multidisciplinary care practitioners required to implement EASE, the increased coordination of resources would not significantly change workflow. This case involved 2 well-resourced tertiary care settings in large cities capable of implementing EASE without additional cost. Of note, no new staff were hired to implement EASE. Instead, grant funding was used to hire research assistants for data collection.

## Conclusions

In the setting of acute abdominal surgery, elder-friendly approaches to care have been associated with reduced health care system costs and were not associated with QALYs. Patients and their caregivers were also less likely to spend money on health services after EASE interventions. This intervention should be implemented broadly. In locations that do not already have the capacity to implement this intervention, costs to implement should be weighed against the estimated costs avoided.

## Notes

Supplement. **eTable. **Resource Use Inventory Costing **eFigure. **Density Plots of Quality-Adjusted Life-Years and Costs per Participant
