# Supplementary material for: Cost-effectiveness Analysis of the Elder-Friendly Approaches to the Surgical Environment (EASE) Intervention for Emergency Abdominal Surgical Care of Adults Aged 65 Years and Older
Source: JAMA Netw Open. 2020 Apr 3;3(4):e202034. doi: 10.1001/jamanetworkopen.2020.2034 (PMC7125431; doi:10.1001/jamanetworkopen.2020.2034)
Supplement: Supplement. — eTable. Resource Use Inventory Costing eFigure. Density Plots of Quality-Adjusted Life-Years and Costs per Participant [file jamanetwopen-3-e202034-s001.pdf]

Supplementary Online Content

Hofmeister M, Khadaroo RG, Holroyd-Leduc J, et al. Cost-effectiveness analysis of the Elder-Friendly Approaches to the Surgical Environment (EASE) intervention for emergency abdominal surgical care of adults aged 65 years and older. *JAMA Netw Open*. 2020;3(4):e202034. doi:10.1001/jamanetworkopen.2020.2034

eTable. Resource Use Inventory Costing

eFigure. Density Plots of Quality-Adjusted Life-Years and Costs per Participant

This supplementary material has been provided by the authors to give readers additional information about their work.

© 2020 Hofmeister M et al. *JAMA Network Open.*

eTable. Resource Use Inventory Costing

Description	Cost
Eye glasses	$230.00[1]
Toilet Bars: Toilet arm rests	$65.00[2]
Contact Lenses: Two-week contact lenses, $262.5 per year with 52 weeks per year = $10.10 per pair	$10.10[3]
Toilet Seat: Raised toilet seat/seat elevator	$89.95[2]
Hearing Aids	$1200.00[4]
Tub Transfer Bench	$154.00[2]
Dentures: Dentures, complete, standard, maxillary or upper jaw; and Dentures, complete, standard, mandibular or lower jaw	$1620.00[5]
Shower Bench: Bath/shower chair without back	$79.00[2]
Joint Brace: Knee orthosis to prevent knee flexion	$559.70[6]
Handrails for Shower: Bathtub/shower stall grab bar	$42.34[2]
Elastic Stockings: Thigh high stocking, 20-30mmHg	$126.40[7]
Hospital Bed: Frame costs $1,300; Mattress costs $380; Rails cost $175	$1855.00[8]
Cane: Four-point cane	$45.00[9]
Bed Pads: Mattress overlay, foam cell	$160.00[10]
Crutches: Forearm crutch pair	$120.00[9]
Bed Alarm	$199.95[11]
Walker: Standard, without wheels	$90.00[9]
Urinary Catheter: Intermittent catheter, plastic	$2.22[12]
Restraints: Posey, 2-point	$2.23[13]
Door Alarms	$20.79[14]
Wheelchair: KI Mobility Tsunami AL	$2934.00[15]
Diapers/Pads/Briefs: Disposable adult diapers, medium, fits waist 32” to 44”	$1.12[12]
Lift Chair: Electric	$129.00[16]
Ostomy Supplies: One-piece colostomy/ileostomy pouch, closed end with seal	$5.63[12]
Safety Bars: Bathtub/shower stall grab bar	$42.34[2]
Informal Care	$29.09/hour[17]
Lost Productive Time	$29.09/hour[17]

1. Alberta Blue Cross - Government-sponsored program participants. https://www.ab.bluecross.ca/government/government-programs.php. Accessed August 23, 2018.

2. Alberta Health. AADL Approved Product List - Small bathing and Toileting Equipment. July 2017.

3. Prescription Contact Lenses. ACUVUE® Brand Contact Lenses. https://www.acuvue.ca/why-contact-lenses/types-of-contacts. Accessed August 23, 2018.

4. Alberta Health. AADL Approved Products List - Hearing Aids, Bone Anchored Hearing Devices and Cochlear Implants. March 2016.

5. Alberta Dental Association and College. Guide for Dental Fees for General Dentists. January 2018.

© 2020 Hofmeister M et al. *JAMA Network Open.*

6. Alberta Health. AADL Approved Product List - Orthotic Benefits. August 2018.

7. Alberta Health. AADL Approved Products List - Ready made Compression Garments. October 2017.

8. Alberta Health. AADL Approved Products List - Homecare Bed, Mattress and Accessories. December 2015.

9. Alberta Health. AADL Approved Products List - Walking Aids and Accessories. July 2017.

10. Alberta Health. AADL Approved Products List - Mattress Overlays, Transfer Aids and Accessories. October 2017.

11. Wander Alert Bed Exit Alarm. www.alzstore.ca. https://www.alzstore.ca/product-p/c243.htm. Accessed August 23, 2018.

12. Alberta Health. AADL Approved Products List - Medical Surgical Supplies. December 2017.

13. Vitality Medical. Posey Economy Limb Holders. https://www.vitalitymedical.com/posey-economy-limb-holders.html. Published 2018. Accessed August 23, 2018.

14. Amazon Canada. SABRE Wireless Home Security Door Window Burglar Alarm. amazon.ca. https://www.amazon.ca/SABRE-Wireless-Security-Window-Burglar/dp/B00M30SKM0. Published 2018. Accessed August 23, 2018.

15. Alberta Health. AADL Approved Product List - Wheelchairs: Manual and Power. October 2017.

16. Lucanus Corporation. Stair Lifts Canada. http://www.lucanus.ca/Stair_Lifts_Canada.html. Accessed August 23, 2018.

17. Alberta Government. 2017 Alberta Wage and Salary Survey. https://work.alberta.ca/documents/wage-and-salary-survey-overview.pdf. Accessed August 23, 2018.

© 2020 Hofmeister M et al. *JAMA Network Open.*

eFigure. Density Plots of Quality-Adjusted Life-Years and Costs per Participant

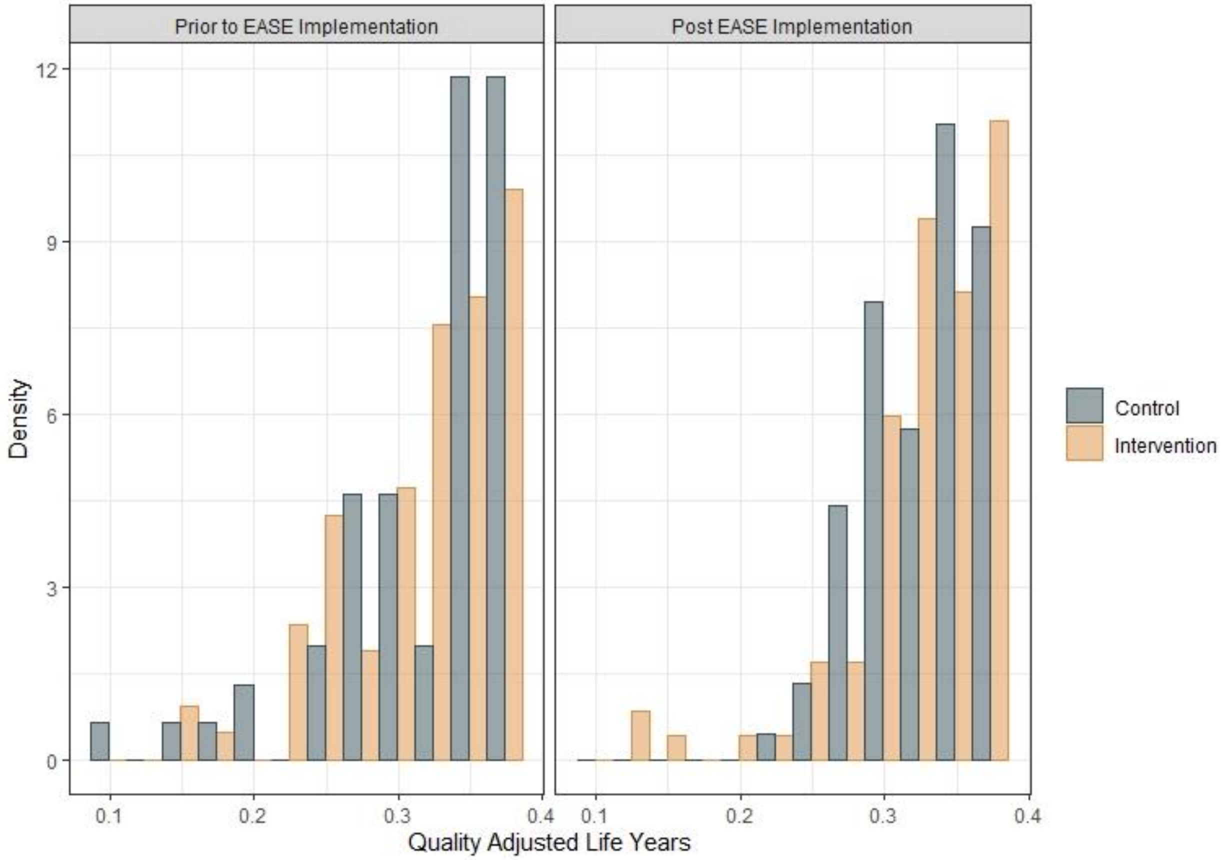

© 2020 Hofmeister M et al. *JAMA Network Open.*

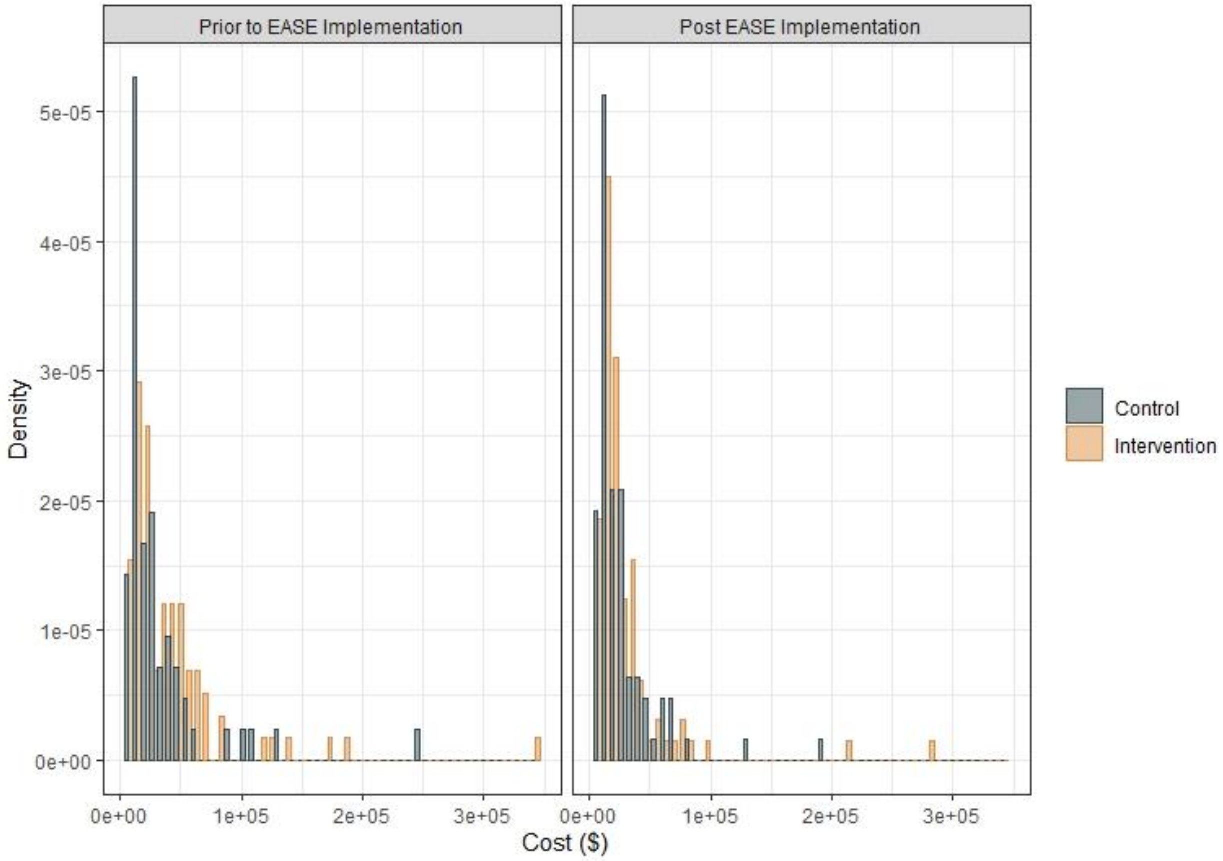

© 2020 Hofmeister M et al. *JAMA Network Open.*
